# The Communicative Modes Analysis System in Psychotherapy From Mixed Methods Framework: Introducing a New Observation System for Classifying Verbal and Non-verbal Communication

**DOI:** 10.3389/fpsyg.2019.00782

**Published:** 2019-04-10

**Authors:** Luca Del Giacco, Silvia Salcuni, M. Teresa Anguera

**Affiliations:** ^1^Department of Social Psychology and Quantitative Psychology, University of Barcelona, Barcelona, Spain; ^2^Department of Developmental Psychology and Socialization, University of Padua, Padua, Italy; ^3^Institute of Neurosciences, Faculty of Psychology, University of Barcelona, Barcelona, Spain

**Keywords:** psychotherapeutic communication, verbal and non-verbal communication, performative language, observation system, mixed methods approach

## Abstract

Communication represents the core of psychotherapy. The dynamic interaction between verbal and non-verbal components during patient-therapist exchanges, indeed, promotes the co-construction of meanings bringing about change within a process of reciprocal influence of participants. Our paper aims to illustrate the building of a new observational instrument of the therapeutic discourse, the Communicative Modes Analysis System in Psychotherapy (CMASP), and its reliability study from Mixed Methods framework. The CMASP is a single classification system analyzing the communication features within therapeutic exchanges. Born to overcome the limits of traditional psychotherapy research which considers verbal and non-verbal dimensions of communication as in polar opposition, the CMASP building was based on the performative function derived from the Speech Act Theory. We used this function as a comprehensive theorization to interpret the communication components in psychotherapy as an integrated and interacting system. In fact, the instrument detects and classifies, at the overall and dimension level, the verbal and extra-linguistic components of psychotherapeutic communication implemented by the therapist and patients in the form of communicative modes. From the observational methodology framework, it was built an instrument able to record and analyze verbal, vocal and interruption behaviors by combining elements of qualitative and quantitative research approaches. The sample consisted of 30 psychotherapy audio recordings and verbatim transcripts of psychotherapy sessions (for a total of 8327 speaking turns). Four main dimensions were elaborated (Verbal Mode-Structural Form, Verbal Mode-Communicative Intent, Vocal Mode, and Interruption Mode) according to the agency role of communication components. The instrument is a field format combined with category systems. For each dimension, we built a category system that is exhaustive and mutually exclusive. From all dimensions, we have a total of 33 categories. Intra-and inter-judge reliability among four independent judges was computed on a total of 503 speaking turns coded through Cohen’s κ and Krippendorff’s canonical agreement coefficients (Cc), respectively. The CMASP showed high intra-and inter-judge agreement at the global, dimensional, and categorical level providing researchers and professionals with a single and flexible classification system, able to give multiple and concurrent information about the psychotherapy process.

## Introduction

Psychotherapy, as an asymmetric help-relationship focused on the patient, represents an experience of sharing and communication (Molina et al., 2013). During psychotherapy session, therapist and patient implement a specific type of communication in the form of therapeutic conversation, as mutual research and exploration through dialogue (Soares et al., 2010). Participants, through language, co-construct meanings that continually evolve promoting the change (Soares et al., 2010; Dagnino et al., 2012). Speech content (verbal dimension) and the different channels conveying it (non-verbal dimension) are the core ingredients of communicative exchanges (Ephratt, 2011). Nevertheless, Weick (1968) claims that linguistic content, in the form of verbal behaviors, constitutes only a small portion of human communication and most of it rests on extra-linguistic behaviors. In particular, voice and interruption behaviors (Mahl, 2014) are important indicators of the underlying psychological processes in communicative exchanges (Weick, 1968).

Historically, verbal and non-verbal components of psychotherapeutic communication have been considered and studied separately, as though they were independent and in polar opposition to each other, leading to the development of separated theorizations and investigations (Westland, 2015). Nevertheless, recent research underlines the need for an integrated communication approach since verbal and non-verbal behaviors are co-occurring and interrelated phenomena that show mutual influences (Jones and LeBaron, 2002). As Jones and LeBaron (2002) claim, “Mutual influence is especially complex and subtle in face-to-face situations because visible forms of communication occur simultaneously with one another and with vocal messages, and exchanges among persons can occur both sequentially and instantaneously” (p. 512). Therefore, the study of mutual influence represents a focal point in comprehending the interpersonal communication in psychotherapy, justifying the integration of verbal and non-verbal components.

The integrated system reflects the complexity of the therapeutic relationship in which verbal and non-verbal dimensions influence each other and interact regulating its co-construction, although they are separate components (Westland, 2015). Precisely, their interaction determines the building of *therapeutic discourse*, a specific type of conversation with an asymmetric structure in which the mutual influence of verbal and non-verbal communication affects the intersubjective processes implemented by both participants (Leahy, 2004; Westland, 2015).

Traditional psychotherapy research, focusing on either verbal or non-verbal communication through separate theories, impedes to bridge these dimensions and to deepen the processes underlying their reciprocal influence. To overcome such a limit, in agreement with the convergence process of natural and human science (Damasio et al., 2001), we derived the performative (or pragmatic) function of the Speech Act Theory (SAT; Searle, 2017) from the linguistic research to explain how verbal and non-verbal dimensions in psychotherapy influence each other despite being separate, underlining the need for an integrated system. Precisely, compared to scholars who based their investigations on this function to study specific aspects of psychotherapeutic communication (e.g., Valdés et al., 2010; Tomicic et al., 2011; Talia et al., 2014), we assumed the performative function as the global theory to describe the mutual influence of communication components within an interactive system, emphasizing the essential role of integration in analyzing the therapeutic actions.

In line with the performative function, language is a part of reality and not its reflection; it represents a tool to perform actions according to which *by saying something, we do something* that in psychotherapy is an aspect connected to change (Krause et al., 2006; Reyes et al., 2008). Such a function integrates the traditional concept of language as merely constative (or propositional) and overcomes the notion of communication as a mechanic process of encoding-transmission-decoding of messages in which sender and receiver represent the ends of the process itself (Ellis and McClintock, 1990).

Within a process of mutual regulation turn-by-turn (*interactive communication*), which can be studied objectively through systematic observation, a speaker who expresses a speech performs an action that is something different from the act of saying *per se* and in which verbal and non-verbal messages interplay conveyed through different sensory systems. Verbal and non-verbal dimensions have an impact on the listener of communication who, in turn, decodes them and implements a communicative act that affects the first speaker (Jones and LeBaron, 2002; Sbisà, 2009).

During the psychotherapeutic encounter, the patient and therapist share and influence their reciprocal internal realities by transmitting information (contents) through recordable verbal behaviors. These verbal messages are expressed in the form of propositional acts (that is to refer and predicate) connected to both the speaker’s communicative intent and the object of the therapeutic work (Arístegui et al., 2009; Valdés et al., 2010). Therefore, within a reciprocal coding and decoding process by both participants, each linguistic act has an impact on the recipient of communication determining, on the one hand, the mutual regulation and co-construction of meanings through conversational sequences and, on the other hand, changes in the internal representations of each participant (Arístegui et al., 2009).

However, each speech emitted is influenced by reciprocal prosodic modulations implemented by patient and therapist during the therapeutic interaction (*intersubjective approach to voice*; Campbell, 2007) and changes in the emotional state of each participant are affected by mutual and observable variations in communicative exchanges, according to the principles of universal recognition of emotions (Thompson and Balkwill, 2006; Tomicic et al., 2011, 2015b). Voice quality and its acoustic parameters (tone, intensity, duration, and timbre) influence the co-construction of meanings by transmitting psychological meanings and emotional messages apart from the verbal content, but verbal and vocal dimensions feel the effects of each other’s action (Jones and LeBaron, 2002; Tomicic et al., 2011). The integration of vocal dimension to speech content is at the basis of regulatory behaviors as any experience of therapeutic interaction (Jones and LeBaron, 2002). Patient and therapist implement a mutual regulation process in the form of coordination sequences of vocal behaviors which are connected to change (Tomicic et al., 2011). Precisely, this process determines a reciprocal influence in the internal organization of both participants and transforms the individual internal functioning in a more complex state (Campbell, 2007; Tomicic et al., 2015b).

Communicative exchanges in psychotherapy, as every kind of human communication, are organized in a speaking turn alternation that patient and therapist can influence through reciprocal interruptions (Li et al., 2005). They represent linguistic acts supplied with intentionality (Wallis and Edmonds, 2017) that violate the turn-taking rules allowing the interrupter to encroach on speaker’s communicative and elaborative space, supporting or hindering the co-construction of meanings and the communicative relationship (Murata, 1994; Li, 2001; Sacks et al., 2015). Therefore, the communicative intent of the interruption enriches the meaning and strength of the speech emitted by the interrupter through the mutual influence with the other verbal and non-verbal dimensions that constitute the speech itself (Jones and LeBaron, 2002). At the same time, within a mutual coding and decoding process, these non-verbal interactive behaviors (Mahl, 2014) impact on the speech of the one who is interrupted producing changes in the interactive dynamics between verbal and non-verbal components (Jones and LeBaron, 2002). Thus, interruptions orient the mutual regulation of participants through coordination sequences that influence the co-construction of meanings and therapeutic discourse (Van Eecke and Fernández, 2016).

According to this performative model of communication, “People not only utilize structural forms, but they also co-construct and negotiate meanings and rules in their ongoing interactions” (Jones and LeBaron, 2002, p. 504). Hence, the interplay of verbal and non-verbal dimensions increases the complexity of communicative exchanges in psychotherapy through mutual influence and regulation processes arising during patient-therapist interactions. The co-occurrence of these communication components models the co-construction of meanings and the unfolding of the therapeutic dialogue pointing out the need for integration.

These processes can be best studied through systematic observation because it represents the most appropriate method to capture the reality of communication exchanges and components in the natural context of the therapeutic setting (Anguera et al., 2018b). Therefore, we need observational instruments for recording and analyzing behaviors which can integrate verbal and non-verbal dimensions and fill the gap of the existing literature (such as the one we are about to introduce), since none of the present tools can keep the components of communication together.

Over the years, various research lines developed around the therapeutic intervention respectively focusing on psychotherapy manualization (e.g., Craske and Barlow, 2006), non-specific factors of change (e.g., Krause, 2005), and psychotherapy process and outcome (e.g., Wampold, 2005), while psychotherapeutic communication area has received less attention (Valdés et al., 2010). However, many scholars (e.g., Bucci, 2007; Lepper, 2009, 2015; Valdés et al., 2010; Tomicic et al., 2011; Weiste and Peräkylä, 2014; Buchholz and Reich, 2015) support the importance of studying the communicative patterns, especially in successful psychotherapeutic encounters, underlining their fundamental role in comprehending patient-therapist interactions.

During the decades, a wide variety of methods arose to study the intersubjective processes between patient and therapist, often involving problems in the field of methodology which increased the complexity and difficulty of studying communication in psychotherapy (Lepper, 2009, 2015). Nevertheless, systematic observation proved to be as the best way to analyze these communication processes.

Scholars in this field have developed various observational tools to analyze verbal and extra-linguistic components of communication in psychotherapy, but they are based on separate theorizations of the communicative dimensions and are not exempt from limits. For example, the Comprehensive Psychotherapeutic Interventions Rating Scale (CPIRS), developed by Trijsburg et al. (2004), considers only the classification of interventions implemented by a therapist resulting from the analysis of common factors to the main psychotherapy orientations (client-centered, group psychodynamic, behavioral, cognitive and systemic orientations). The Client Behavior System (CBS), developed by Hill et al. (1992) as a revised version of the Client Verbal Response Category System (CVRCS; Hill et al., 1981), focuses in particular on patient’s verbal behaviors, distinguishing eight nominal and mutually exclusive categories derived from different theoretical perspectives. Finally, the Therapeutic Activity Coding System (TACS-1.0), developed by Valdés et al. (2010), is a single system based on the notion of performative language which classifies only verbal communicative actions of patient and therapist by micro-analyzing each speaking turn during relevant episodes of the psychotherapy process.

As for voice and interruptions in psychotherapy, research is not as extensive (e.g., Weiste and Peräkylä, 2014; Buchholz and Reich, 2015; Oka et al., unpublished) as the research on verbal communication. Observational systems to classify voice in the psychotherapeutic context are not so many, while those to observe interruptions are not present, to our knowledge. With regard to the study of voice, for example, the Client Vocal Quality (CVQ) and Therapist Vocal Quality (TVQ) are two classification systems developed by Rice and Kerr (1986) to separately detect the client’s vocal style in any given utterance and the therapist’s vocal qualities affecting the client’s participation in the therapeutic work, apart from speech content. Finally, the Vocal Quality Pattern (VQP) was developed by Tomicic et al. (2015a) as a single coding system to classify patient and therapist’s vocal quality, apart from the content of speech considering specific acoustic parameters of voice, during relevant episodes of the psychotherapy process. Such a system includes four vocal quality patterns (Reporting, Connected, Affirmative, Introspective, and Emotional) and three non-coding categories of vocal patterns, but it does not distinguish the positive and negative emotions of speech. Referring to the study of interruptions, systems for classifying this kind of behaviors are not traceable in psychotherapy framework. In psychotherapy research as well as in intersubjectivity and self-regulation models, distinct detection of positive and negative emotions and interruption behaviors is extremely important because they affect the change and psychotherapy process (Carver and Scheier, 1990; O’Reilly, 2008; Stalikas and Fitzpatrick, 2008; Schutte, 2013).

Although all these classification systems contribute to studying the communicative components of the therapeutic discourse, they do not consider the mutual influence of verbal and non-verbal dimensions and often focus on a specific participant or aspect of the communicative exchange. Moreover, although some classification systems are built as single systems to analyze speech of both therapist and patient, they do not go deep in the study of some aspects of communication (for example, the VQP includes only the Emotional category, not distinguishing between positive and negative emotions, or emotions with and without verbalizations). Furthermore, they often may segment a speaking turn to micro-analyze the communicative behaviors but not providing information at a more global level (e.g., TACS-1.0; Valdés et al., 2010). Finally, as we mentioned previously, there is a lack of systems for classifying interruptions in psychotherapy.

To overcome these limitations, we consider the need for a comprehensive classification system able to study and describe verbal and extra-linguistic behaviors implemented reciprocally by patient and therapist turn-by-turn during communicative exchanges. Furthermore, this system must be able to understand the mutual influence and evolution of such communicative behaviors during psychotherapy. For these reasons, inspired by an interdisciplinary perspective (Damasio et al., 2001) and starting from the performative function of language (Searle, 2017), we have developed -within an exploratory and descriptive design- the Communicative Modes Analysis System in Psychotherapy (CMASP), that we introduce in this paper.

The CMASP is born as an attempt to solve the problem of studying communication in psychotherapy according to a comprehensive theory. It has been developed to be a single and flexible observational system able to detect and classify (together or separately) both verbal and extra-linguistic components of communication expressed by the therapist and patient during the therapeutic exchange. Furthermore, the instrument allows identifying a communication profile for each participant and their interaction by integrating the communicative modes implemented. It provides valuable support in increasing knowledge about patient-therapist exchanges by detecting the communicative profiles able to build change during the psychotherapy process, and this is impossible using existing tools.

To describe patient-therapist communicative interactions and to analyze their mutual influence at the verbal and extra-linguistic level, the CMASP building is based on the performative function of language (Searle, 2017), which is connected to change in psychotherapy (Krause et al., 2006; Reyes et al., 2008), combined with Campbell’s theorization (Campbell, 2007) and the principles of universal recognition of emotions (Thompson and Balkwill, 2006). Moreover, its constituent categories are derived from previous works adapted to the goals of our investigation (Hill, 1978; Goldberg, 1990; Stiles, 1992; Murata, 1994; Li, 2001; Valdés et al., 2005, 2010; Krause et al., 2009; Tomicic et al., 2015a) and from the building process of the classification system itself.

Specifically, as a single system, the CMASP permits a rigorous and systematized analysis of verbal and non-verbal communicative modes implemented by both patient and therapist in each speaking turn during the psychotherapeutic discourse. All this allows realizing comparative and sequential analyses which provide knowledge of the participants’ mutual interaction process, the way communication evolves, and the communicative actions which affect the change during the psychotherapeutic process.

In recent years, a growing interest in integrating qualitative and quantitative methods has been developing in psychotherapy research. This integration provides a more comprehensive view of the patient-therapist interaction as it is supported by objective measures through a complementary perspective (Lutz and Hill, 2009), the search for mixed methods, which offers both rigor and flexibility in approaching the reality of the therapeutic relationship (Anguera and Hernández-Mendo, 2016; Anguera et al., 2018a).

The purpose of this paper is, firstly, to introduce the building of the CMASP by describing the methodology used to realize it and showing its ability in detecting and coding multiple aspects of communication in psychotherapy through its constituent dimensions and categories. Secondarily, we would present its first reliability psychometrics, for both inter-and intra-rater values, and its applications in the form of descriptive statistics of the subscales trend and an example of coding.

## Materials and Methods

The CMASP is founded on the systematic observation (Anguera et al., 2001) of verbal, vocal and interruption behaviors in patient-therapist communicative exchanges; this methodology, in turn, is based on a mixed methods approach (Plano Clark et al., 2015) integrating qualitative (QUAL) and quantitative (QUANT) data according to an exploratory sequential design (Fetters et al., 2013). Therefore, in line with a non-participant and indirect observation of natural language (Anguera et al., 2018b) within the ecological and not structured context of the therapeutic setting, patient and therapist’s communicative behaviors were subjected to qualitative and quantitative analyses. In particular, verbal behaviors were converted into documentary material to analyze the content of each speech; to analyze vocal and interruptions behaviors, the acoustic characteristics of speech and the impact of these on the listener of the patient-therapist communicative exchanges were observed through a careful listening of therapeutic session recordings, apart from the content of messages. Although this methodology is intensive and implies working with a reduced number of participants, it permits the collection of a large number of records with high rigor (Castañer et al., 2016, 2017; Arias-Pujol and Anguera, 2017; García-Fariña et al., 2018; Rodríguez-Medina et al., 2018; Suárez et al., 2018) through the use of an observational instrument (the CMASP in this research).

Mixed methods research represents “a new movement, or discourse, or research paradigm (with a growing number of members) that has arisen in response to the currents of qualitative research and quantitative research” (Johnson et al., 2007, p. 113). The concepts and technicalities of quantification and data transformation are a recurrent theme in works written by eminent figures in the field of mixed methods research (Sandelowski, 2001; Creswell et al., 2003; Bazeley, 2009, 2018; Sandelowski et al., 2009; Onwuegbuzie et al., 2018; Schoonenboom et al., 2018). Several options are possible, and we select that one more suitable, considering the qualitative nature of data.

Quantification in observational methodology (in this study performed by using the CMASP) is particularly robust because, apart from simple frequency counts, contemplates other essential primary parameters, such as order and duration (Bakeman, 1978; Anguera et al., 2001; Bakeman and Quera, 2011; Quera, 2018), thereby providing the researcher with the means to map the different components of a behavior as it occurs.

In observational methodology, primary parameters are frequency, order, and duration; they are structured in the form of levels that follow a *progressive order of inclusion* (Anguera and Blanco-Villaseñor, 2003) according to which the corresponding data progressively acquire greater power. In particular, frequency provides the least information, while order gives information on both frequency and sequence of behaviors; finally, duration supplies information on frequency and order by adding the number of time units for each occurrence of a behavior.

The specific consideration of the order parameter is crucial for detecting hidden structures through the quantitative analysis of relations among different codes in systematized observational datasets. Precisely, since the initial dataset -deriving from a notably rich qualitative component- contains information on the order, it can be analyzed using a wide range of quantitative techniques working with categorical data (e.g., lag sequential analysis, polar coordinate analysis, and detection of T-Patterns) and producing a set of quantitative results which are then qualitatively interpreted, bringing about a seamless integration. Therefore, such quantitative techniques aim at searching invisible structures and studying how these evolve.

According Creswell and Plano Clark (2011), “there are three ways in which mixing occurs: merging or converging the two datasets by actually bringing them together, *connecting the two datasets by having one build on the other*, or embedding one data set within the other so that one type of data provides a supportive role for the other data set” (p. 7; the emphasis is our). Just based on the second option (*Connecting*) of integration of qualitative and quantitative elements, we perform this connection starting from systematic observation and transforming usual qualitative data of records in another dataset (here recorded by the CMASP). This last one allows including the record parameters of order and duration, being possible to obtain a matrix of data which are analyzable through quantitative techniques (Anguera et al., 2018b). Each session record will generate a matrix of codes (generally not regular) in the dataset, and each row will express the co-occurrences (corresponding to the various dimensions) carried out in each of the successive units.

The wide range of opportunities, available for processing data derived from observation, supports the idea that purely observational studies should be considered as mixed methods research studies (in which *connecting* represents an integration form implying to quantitize the qualitative records), even though they constitute a special case and do not follow traditional patterns (Anguera et al., 2017).

### Design

**FIGURE 1:**
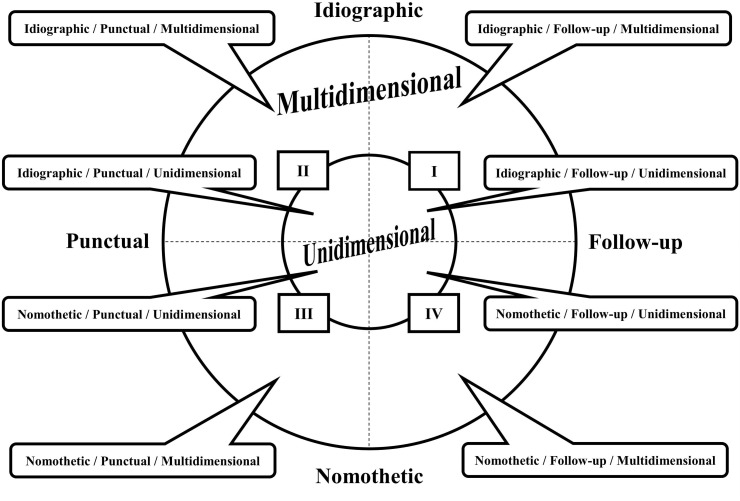
Representation of the observational designs (adapted from Blanco-Villaseñor et al., 2003, p. 115). The intersection of the three dichotomous criteria (the unit of study, the continuity of recording, and the number of dimensions) brings about eight possible combinations, corresponding to the eight observational designs distributed in the four quadrants.

Within the mixed methods perspective, the observational design (Blanco-Villaseñor et al., 2003) represents an empirical model of organization of the study connected to the research aims and in line with the systematic observation used which lead the decisions about data collection, organization, and analysis. The intersection of three dichotomous criteria (the unit of study, the continuity of recording, and the number of dimensions) provides eight different observational designs distributed in four quadrants (Figure 1).

The *unit of study* is divided into the Idiographic option (one unit corresponding to one participant or various participants with a stable bond) and Nomothetic option (different units). The *continuity of recording* is divided into the Punctual option (one session recorded) and Follow-up option (different sessions recorded over time). This last one, in turn, can be specified in inter-sessional (the recording obtained along different sessions) and intra-sessional (the recording obtained from the beginning to the end of a session). Finally, the *number of dimensions* is divided into the Unidimensional or Multidimensional options, depending on the number of response levels considered and connected to the study aims. On several occasions, one or more dimensions can be developed in subdimensions (Blanco-Villaseñor et al., 2003).

Given the complexity of this study, the most suitable observational design, among those involving a low level of intervention (Chacón-Moscoso et al., 2014), was the Nomothetic/Follow-up/Multidimensional (N/F/M; Anguera and Izquierdo, 2006) included in the Quadrant IV of the systematic observation designs representation as it presents the most wealth of information and a higher complexity (Figure 1; Blanco-Villaseñor et al., 2003). Specifically, the study was *nomothetic* because it was focused on a plurality of units in which different patients, in interaction with the same therapist, were analyzed independently. Moreover, intra-and inter-session analyses were performed, reflecting the *follow-up* recordings. Finally, the evaluation of verbal, vocal and interruption behaviors corresponded to the observation of multiple channels of communication, typical of the *multidimensional* design. As it is possible to notice, there is a full correspondence between the observational design selected for this study and the structure of the CMASP.

### Participants and Material

We developed the CMASP at the Dynamic Psychotherapy Service belonging to the Interdepartmental Laboratories for Research and Applied Psychology (LIRIPAC), a recognized research center of the University of Padua (Italy). The ethics committee of psychology faculty of the University of Padua approved the collection of the research material (informed consents, the audio recording of sessions, and confidentiality modes of procedures) which followed the ethical guidelines and procedures of the LIRIPAC, based on the Italian law about privacy and confidentiality (n. 196/03). We discussed the specific research practice and ethical procedure of this investigation with the Director of the Centre who approved them before the research began in 2016.

We followed the ethical standards for research outlined in the *Ethical Principles of Psychologists and Code of Conduct* (American Psychological Association [APA], 2017). Therefore, we assured confidentiality by replacing the participants’ personal information. As for listening to the audio recordings, we guaranteed confidentiality not providing personal data of the speakers to the trained coders who were in charge of their listening and transcription. We did not award incentives, and we emphasized voluntary participation. In line with the Declaration of Helsinki, we collected the informed consents of the therapist (verbal consent) and each patient (written consent), finalized to research aims, before realizing data collection and audio recording. In other words, we conducted the study after the end of the psychotherapy treatments.

For the CMASP development, we selected 10 weekly individual psychotherapies among those of patients self-referred to the Dynamic Psychotherapy Service (DPS) of the University of Padua. Psychotherapy sessions collection was managed, in respect with patients’ recruitment, according to the following criteria: (a) each patient agreed to participate and signed the informed consent; (b) all participants completed the entire psychotherapeutic assessment phase; (c) each patient, by a previous screening to the assessment, completed the depressive scale of the Beck Depression Inventory-II (BDI-II; Italian version, Ghisi et al., 2006) and the Symptom Checklist 90 Revised (SCL-90-R; Italian version, Sarno et al., 2011), obtaining scores greater than or equal to the 85° percentile and the *T*-score of 60, respectively; (d) the audio recording of each session was complete. Moreover, patients met the following exclusion criteria: (a) absence of psychiatric diagnosis; (b) absence of ongoing pharmacological treatment for depression; (c) absence of previous psychological treatment.

The choice of selecting depressed patients was due to (a) the prevalence of this kind of patient who self-referred; (b) research reasons for obtaining a sample as uniform as possible; (c) the specific communicative features of this kind of patients which represent an expression of their symptoms. In fact, patients with depressive symptoms tend to speak more slowly and monotonously with less volume and voice modulation (Rottenberg and Gotlib, 2004), moreover, they tend to show high variation in prosody connected to the severity of symptoms (Yang et al., 2013).

Patients consisted of 10 university students (5 men and 5 women; age *M* = 26 years, *SD* = 3.91, Min = 22 years, Max = 32 years), residing in urban and rural areas of Italy; all of them were in care by the same female therapist (aged 39 years) with 13 years of expertise in the psychodynamic approach. By the administration of the BDI-II (Italian version, Ghisi et al., 2006) and SCL-90-R (Italian version, Sarno et al., 2011), all the patients showed depressive symptomatology. Specifically, they showed positive scores in the Total Score (*M* = 93.86, *SD* = 7.15, Min = 80, Max = 99), Somatic-Affective Area (*M* = 95.00, *SD* = 2.77, Min = 90, Max = 99), and Cognitive Area (*M* = 94.71, *SD* = 5.74, Min = 85, Max = 99) of the BDI-II. Moreover, they showed positive scores in the Global Severity Index (*M* = 61.14, *SD* = 8.15, Min = 53, Max = 75) and Depression Scale (*M* = 67.86, *SD* = 6.09, Min = 60, Max = 75) of the SCL-90-R. For each patient, the audio recordings (50 min each) and their verbatim transcriptions of the first three psychotherapeutic sessions were considered, for a total of 30 psychotherapy sessions. Afterward, we eliminated one session since it did not satisfy the inclusion criteria (the audio recording interrupted 10 min after the beginning) obtaining a sample of 29 psychotherapy sessions (29 audio recordings and 29 verbatim transcripts). Each transcription and the corresponding audio recording were divided into speaking turns.

To build the CMASP, we drew 3 cases of psychotherapy (each one consisted of 3 sessions) from the 10 cases considered, for a total of 9 sessions. Afterward, we randomly selected and observed audio recordings and their transcriptions of six sessions from the three cases of psychotherapy, for a total of 2095 speaking turns (1048 therapist speaking turns + 1047 patient speaking turns). These 3 cases of psychotherapy were excluded from further analyses, obtaining a definitive sample of 7 cases (4 men and 3 women) for a total of 6232 speaking turns (3121 therapist speaking turns + 3111 patient speaking turns). Finally, two sessions and their audio recordings, for a total of 503 speaking turns (252 therapist speaking turns + 251 patient speaking turns), were randomly selected among the remaining 20 sessions to perform data quality control.

#### Judges and Training Process

Three undergraduates and one Ph.D. students in psychology were recruited as judges and trained for the CMASP. Training consisted of 3-h classes 3 times a week (for a total of 35 h). During such a period, the judges learned the verbatim transcription norms as well as the usage of the Audacity^®^ recording and editing software (version 2.2.1; Audacity Team, 2017) for observing and coding the audio recordings. Moreover, they studied the coding and training manual of the CMASP (Del Giacco et al., 2018) as well as they done exercises -rating 11 extracts of psychotherapy sessions audio recordings and transcripts for a total of 550 speaking turns coded (275 therapist speaking turns and 275 patient speaking turns)- and participated in discussion groups about encodings attributed.

### Instruments

In systematic observation, recording instruments (e.g., to record and coding data) and observation instruments (that is purpose-designed *ad hoc* instruments) are differentiated (Anguera et al., 2018b).

#### Recording Instruments

Each 50-min therapeutic session was recorded in the therapist’s room through an MP3 audio recorder, positioned at an equal distance from the therapist and patient to reduce and control reactivity biases. Trained undergraduates realized a verbatim transcription for each audio recording of psychotherapy sessions to observe verbal behaviors during patient-therapist communicative exchanges. Moreover, they used the Audacity^®^ recording and editing software (version 2.2.1; Audacity Team, 2017) to perform the extra-linguistic behaviors observation. Such software is a support instrument to listen to audio tracks which shows the sound wave and enables the observer to stop, segment, trace, and code the audio recording for applying the categories according to the coding manual. The dataset was built using Excel.

Data quality control analyses were performed through the Tool for the Observation of Social Interaction in Natural Environments (HOISAN, v. 1.6.3.3.4; Hernández-Mendo et al., 2012) and Sequential Data Interchange Standard-Generalized Sequential Querier computer program (SDIS-GSEQ, v. 4.1.3; Bakeman and Quera, 2011). Finally, descriptive statistics were performed through SPSS v. 23.0 Statistics statistical software.

### Procedure

#### Development of the CMASP

The CMASP was elaborated within the observational methodology framework as an *ad hoc* indirect observation system of the therapeutic discourse (Anguera et al., 2018b) able to detect, record and classify verbal, vocal and interruption behaviors implemented turn-by-turn by patient and therapist, in the first phases of psychotherapy.

Based on this type of observation, the instrument building took place by implementing a recurrent process which oscillated between the observation of psychotherapeutic reality through audio recordings and transcripts and the theoretical framework that supporting the knowledge of that reality. To this end, the CMASP derived from the combination of two main instruments of the observational method, the field format and category systems, which were elaborated *ad hoc* for this specific observational study, exploiting the advantages of each to understand the reality of the therapeutic dialogue. Their combination rests on the theoretical framework of the observed reality and provides the instrument with the flexibility and dimensionality of the field format as well as with the consistency of the category systems (Anguera et al., 2007, Anguera et al., 2018b).

In the CMASP building, the recording process -leading up to a systematized recording of verbal and extra-linguistic behaviors with maximum external control- was divided into two different phases: the exploratory or passive phase (pre-scientific) and the active phase (scientific; Anguera et al., 2007). These phases were realized using the audio recordings of six sessions randomly selected from the three cases of psychotherapy previously drew.

During the pre-scientific phase, firstly we defined the structural criteria of the observation tool starting from the theoretical framework of the performative function of verbal and non-verbal behaviors (Krause et al., 2006; Reyes et al., 2008; Searle, 2017), reciprocally performed by patient and therapist through speech to co-construct the communicative relationship and meanings. The criteria were deduced after an analysis of the characteristics of communication in psychotherapy from related scientific literature and the variables studied in other research paper. To this end, we have carried out a review of databases (Google Scholar, Scielo, Dialnet, PsycINFO, PsycARTICLES, PsycCRITIQUES, and PubPsyc) using the following keywords: “verbal communication and performative language”; “non-verbal communication and performative language”; “psychotherapy and communication and performative language”; “psychotherapy and Speech Act Theory.” We reviewed the abstracts and papers to select the studies related to the analysis of communication components according to the performative function of language (Hill, 1978; Goldberg, 1990; Stiles, 1992; Murata, 1994; Li, 2001; Valdés et al., 2005, 2010; Krause et al., 2009; Tomicic et al., 2015a). After discussing a preliminary list, we established core criteria and their definitions characterizing four dimensions: Verbal Mode-Structural Form (VeM-SF), Verbal Mode-Communicative Intent (VeM-CI), Vocal Mode (VoM) and Interruption Mode (IM). In particular, two dimensions were defined to analyze verbal behaviors: the VeM-SF, concerning the propositional content and corresponding to the structure by which speech expressed the communicative mode; the VeM-CI, concerning the performative content and corresponding to the communicative intent of the speaker’s speech.

In this exploratory phase, three audio recordings were chosen at random from the six sessions so that they respectively corresponded to the first, second and third session of different individual psychotherapies. These audio recordings were listened through Audacity^®^ software (version 2.2.1; Audacity Team, 2017) and verbatim transcribed. Such a step was fundamental for improving the training to observation, reducing biases (e.g., reactivity or expectation biases), as well as defining the norms for verbatim transcription, and elaborating a narrative recording (that is the first description of behaviors observed in the natural context with little constraints; Anguera et al., 2018b) at the root of the systematic observation process of communication.

To realize the narrative recording and observing verbal and extra-linguistic behaviors, we first unitized verbatim transcriptions and audio recordings in line with Krippendorff’s (2013) procedures; they were structured in text blocks and audio blocks, respectively. We defined a text block as the whole speech in the transcript included between the opening and closing sentences of each therapy session. The audio block corresponded to that of the transcription, and it was marked in the audio recording through Audacity^®^ software (version 2.2.1; Audacity Team, 2017). Afterward, we organized the text and audio block in speaking turns according to patient and therapist’s communicative exchanges. One speaking turn corresponded to the piece of speech emitted by one speaker from the moment he/she began to speak until the other speaker took the floor. Given the correspondence between the audio and text block, we marked the speaking turn in the audio recording through Audacity^®^ software at the change of speaker (therapist or patient) who emitted the speech (Tomicic et al., 2011).

We assumed the speaking turn as the unit of analysis of communicative exchanges, and it was equivalent in both the transcription and the audio recording. To facilitate a microanalytical observation and to perform subsequent comparative analyses, each transcript and audio recording was divided into ten segments according to the procedure defined by Colli et al. (2014) for the Collaborative Interaction Scale-Revised (CIS-R). This choice permitted to obtain the same number of pairs of therapist–patient turns in all the segments as well as it allowed segmenting the CMASP in the same way as other tools for psychotherapy process analysis do (e.g., the CIS-R). Finally, speaking turns were sequentially numbered and named with T and P to differentiate the speech of therapist and patient, respectively.

After carrying out the unitizing process, we observed the audio recordings and transcripts of the psychotherapy sessions and elaborated a list of communicative behaviors for each dimension. Each dimension was exhaustively observed until we detected and listed all possible communicative behaviors that represented the core criterion.

During the scientific phase, we deduced a list of possible categories for each dimension, adapted to the study goals, from the previous works selected. With the list of communicative behaviors for each dimension of the exploratory step, we performed a grouping process around concepts of the theoretical framework characterizing each provisional category. During this process, we improved the definitions and features of each category. Contemporarily, we performed a thematic grouping process of a series of communicative behaviors detecting new categories for each dimension. We defined provisional lists of categories systems that were discussed and modified until we achieved an agreement on each one.

As a result, we obtained a set of exhaustive and mutually exclusive (E/ME) categories of communicative behaviors for each criterion dimension (Anguera et al., 2018b), ensuring a good flexibility degree of the classification system. In other words, within the therapeutic discourse, each speech of patient and therapist could be evaluated according to the four dimensions of the instrument, while each communicative behavior identified could be assigned to one (exclusivity condition) and only one (mutual exclusivity condition) category within the category system of the corresponding dimension (Anguera and Izquierdo, 2006).

Once the categories were defined, an evidence check was performed on three new psychotherapeutic sessions -randomly selected among those of the three cases drew- to verify that new behaviors could not emerge, confirming the exhaustiveness of category systems after the instrument building. In this stage, the manual of the observational instrument (Del Giacco et al., 2018) was developed.

#### Coding Manual

A coding and training manual (Del Giacco et al., 2018) was elaborated to present the organization of the CMASP, the norms for the verbatim transcription, and the explanation of the Audacity^®^ software usage (version 2.2.1; Audacity Team, 2017). Inside it, we described the categories of the CMASP dimensions. We illustrated each category definition through textual (and audio) examples and counter-examples, extrapolated from the observation of verbal and extra-linguistic psychotherapeutic communication, to identify and discriminate verbal, vocal and IMs, respectively. Furthermore, we showed and explained the procedure for unitizing the transcription and its audio recording as well as detecting the minimal unit of analysis for each dimension. For VeM-SF, VeM-CI, and VoM coding, we explained in the manual both the criteria for segmenting each speaking turn when a coder detected multiple categories for one dimension and the norms to be used to annotate these. Steps for coding verbal and extra-linguistic modes in the transcription and audio recording were defined. In the case of speaking turn segmentation due to VeM-SF, VeM-CI, and VoM coding, we described the rules for obtaining a global encoding. This aspect allows realizing comparative and sequential analyses as well as obtaining a systematized record in the form of a dataset (that is systems of codes structured as matrices) in which each speaking turn expressed multiple event codes.

Given the correspondence in the unitizing procedures of verbatim transcription and audio recording, we assumed the former as the coding sheet to note the observation and coding of verbal dimensions and extra-linguistic dimensions, respectively. Afterward, encodings -detected and transcribed for each dimension- were reported in a global coding sheet to obtain multiple event codes for each speaking turn.

#### Rigorous Data Quality Control of the CMASP

After the evidence check, control analyses were implemented through two quantitative statistical techniques to verify and ensure the data quality and the reliability of the instrument. The first one, the intra-observer reliability, was computed through Cohen’s kappa coefficient (κ; Cohen, 1960) to verify the degree to which one observer’s encodings of the same transcript and audio recording remained constant at two different times (in this study, we realized the second coding of the same transcription and audio recording after 1 month). The second one was the inter-observer reliability to verify the agreement level of at least three observers’ encodings of the same transcript and audio recording at the same point in time. It was computed, at the global and dimensional level, through Krippendorff’s canonical agreement coefficient (Cc; Krippendorff, 1980) -an adaptation of Cohen’s kappa- while, at the categorical level, as an average value of all the Cohen’s kappa coefficients (κ; Cohen, 1960) calculated on different couples of observers (all the possible combinations of the four observers). These analyses were performed on the encodings of four judges -trained for the CMASP and its coding procedure (Losada and Manolov, 2015; Anguera et al., 2018b)- who observed 503 speaking turns, corresponding to the material of 2 psychotherapy sessions (1 verbatim transcription + 1 audio recording each one) randomly selected from the seven cases of the definitive sample. Although we observed only two sessions, the number of speaking turns was adequate to consider the material at a microanalytic level.

The four judges realized the coding independently, applying the CMASP on one selected psychotherapy session at a time. An observer chief was selected among the four judges to compute the intra-observer reliability.

Each reliability was computed for the CMASP, at the overall and dimensional level, through HOISAN v. 1.6.3.3.4 (Hernández-Mendo et al., 2012) and, at the categorical level, through SDIS-GSEQ v. 4.1.3 (Bakeman and Quera, 2011).

## Results

Firstly, we present a general description of the CMASP. Afterward, we discuss the reliability study results and, finally, we report the CMASP applications to the sample (descriptive statistics of subscales trend and an example of coding).

### General Presentation of the Classification System

The CMASP is an *ad hoc* classification system for the indirect observation of communication in psychotherapy, as a combination of a field format system for each criterion dimension and category systems, which analyzes (together or separately) patient and therapist’s verbal, vocal and interruption behaviors turn-by-turn.

The instrument consists of four dimensions (VeM-SF, VeM-CI, VoM, IM), two of them referred to two aspects of verbal behaviors and the others related to vocal and interruption behaviors of communication, respectively.

A total of 33 categories describes patient and therapist’s verbal and extra-linguistic behaviors, respectively. Each dimension comprises a set of these categories in the form of exhaustive and mutually exclusive category system, as described below. Each speaking turn can present one and only one communicative mode for each dimension, but it can show co-occurrent communicative modes belonging to different dimensions.

Concerning the analysis of verbal modes, six categories constitute the VeM-SF dimension (Courtesies, Assertion, Question, Agreement, Denial, and Direction), while the VeM-CI dimension consists of eight categories (Acknowledging, Informing, Exploring, Deepening, Focusing, Temporizing, Attuning, and Resignifying). Concerning the VoM dimension, it consists of eight categories (Reporting, Connected, Declarative, Introspective, Emotional-Positive, Emotional-Negative, Pure Positive Emotion, and Pure Negative Emotion). The communicative intent of each category is associated with both a peculiar acoustic parameters combination and specific mode of the speaker’s speech affecting the listener of communication, apart from the verbal content. Moreover, the “emotional” categories (Emotional-Positive, Emotional-Negative, Pure Positive Emotion, and Pure Negative Emotion) are defined and described according to the principles of universal recognition of emotions (Thompson and Balkwill, 2006). Concerning the IM dimension, eleven categories are detected and specified in cooperative, intrusive, neutral and failed interruptions (Cooperative-Agreement, Cooperative-Assistance, Cooperative-Clarification, Cooperative-Exclamation, Intrusive-Disagreement, Intrusive-Floor taking, Intrusive-Competition, Intrusive-Topic change, Intrusive-Tangentialization, Neutral Interruption, Failed Interruption).

These categories are characterized by a description derived from the application of the observational method as well as from the previous works mentioned. Moreover, each definition of the VoM categories is supported by the description of the combination of acoustic parameters associated. Finally, a code for each category is established (for a detailed description, see “Appendix I. Description of the CMASP dimensions and categories”).

### Reliability Study of the CMASP

As shown in Table 1, results obtained at the overall, dimensional and categorical level of the CMASP are all greater than or equal to 0.81 in both psychotherapy session encodings, indicating an almost perfect level of the intra-judge reliability (κ ≥ 0.81; Cohen, 1960). It is possible to notice that some categories are present only in a psychotherapy session but not in the other one (e.g., Courtesies, Cooperative-Assistance); however, their scores show an almost perfect agreement (κ ≥ 0.81) in the session in which they were detected. Finally, some categories are not present since they do not appear in either session (e.g., Direction, Temporizing, Pure Negative Emotion). It does not represent a negative aspect of reliability, but on the contrary, it means that the judge shows a total agreement in not coding these categories in each session at two different times.

**Table 1:** Intra-judge reliability of the CMASP (*N* = 503 speaking turns).

CMASP	1st session (*n* = 220)	2nd session (*n* = 283)	*M*	*SD*
Overall	0.97	0.99	0.98	0.01
Verbal Mode-Structural Form (VeM-SF)	0.97	0.99	0.98	0.01
Courtesies (SF1)	1.00	TANC		
Assertion (SF2)	0.93	0.98	0.96	0.04
Question (SF3)	0.94	0.97	0.96	0.02
Agreement (SF4)	0.93	0.99	0.96	0.04
Denial (SF5)	TANC	1.00		
Direction (SF6)	TANC	TANC		
Verbal Mode-Communicative Intent (VeM-CI)	0.93	0.98	0.96	0.04
Acknowledging (CI1)	0.99	0.99	0.99	0.00
Informing (CI2)	0.87	TANC		
Exploring (CI3)	0.88	0.93	0.91	0.04
Deepening (CI4)	0.70	0.95	0.83	0.18
Focusing (CI5)	0.69	0.95	0.82	0.18
Temporizing (CI6)	TANC	TANC		
Attuning (CI7)	1.00	1.00	1.00	0.00
Resignifying (CI8)	1.00	0.92	0.96	0.06
Vocal Mode (VoM)	0.97	0.94	0.96	0.02
Reporting (VM1)	1.00	TANC		
Connected (VM2)	0.91	0.93	0.92	0.01
Declarative (VM3)	0.96	0.91	0.94	0.04
Introspective (VM4)	0.71	1.00	0.86	0.21
Emotional-Positive (VM5)	0.91	0.90	0.91	0.01
Emotional-Negative (VM6)	0.95	0.66	0.81	0.21
Pure Positive Emotion (VM7)	1.00	1.00	1.00	0.00
Pure Negative Emotion (VM8)	TANC	TANC		
Interruption Mode (IM)	0.91	0.96	0.94	0.04
Cooperative-Concurrence (IM1)	0.95	0.97	0.96	0.01
Cooperative-Assistance (IM2)	TANC	1.00		
Cooperative-Clarification (IM3)	0.83	0.95	0.89	0.08
Cooperative-Exclamation (IM4)	TANC	1.00		
Intrusive-Disagreement (IM5)	1.00	1.00	1.00	0.00
Intrusive-Floor taking (IM6)	TANC	0.91		
Intrusive-Competition (IM7)	TANC	1.00		
Intrusive-Topic change (IM8)	TANC	TANC		
Intrusive-Tangentialization (IM9)	TANC	TANC		
Neutral interruption (IM10)	0.94	0.80	0.87	0.10
Failed Interruption (IM11)	TANC	0.89		


*TANC, Total Agreement in the Not Coded Category; the intra-judge reliability was computed through Cohen’s kappa (κ); κ: insufficient (lower than or equal to 0.60), substantial (between 0.61 and 0.80), satisfactory (greater than or equal to 0.81).*

As we mentioned above, the inter-judge reliability was computed, at the global and dimensional level, through Krippendorff’s Cc and, at the categorical level, as an average value of all the Cohen’s kappa coefficients derived from the four judge’s encodings of the two psychotherapy sessions considered (220 and 283 speaking turns, respectively), for a total of 503 speaking turns coded. As shown in Table 2, results obtained at the overall and dimensional level of the CMASP are percentages greater than or equal to 81%, indicating an almost perfect level of the inter-judge reliability (Cc ≥ 81; Krippendorff, 1980). At the categorical level, percentages show an inter-judge agreement level which varies between substantial (61% ≤ κ ≤ 80%) and almost perfect (κ ≥ 81%; Cohen, 1960). The categories detected by computing the intra-judge reliability also appear in one session, but not in the other one, by the inter-judge reliability computation. These categories present an agreement level varying between substantial (61% ≤ κ ≤ 80%) and almost perfect (κ ≥ 81%) in the session in which they were detected. Finally, the same categories not detected by computing the intra-judge reliability computation neither appear by the inter-judge reliability computation. Here again, this expresses a total agreement by the four judges in not coding these categories in either psychotherapy session.

**Table 2:** Inter-judge reliability analysis of the CMASP (*N* = 503 speaking turns).

CMASP	1st session (*n* = 220)	2nd session (*n* = 283)	*M*	*SD*
Overall	93^∗∗^	94^∗∗^	93.50^∗∗^	0.71^∗∗^
Verbal Mode-Structural Form (VeM-SF)	95^∗∗^	95^∗∗^	95.00^∗∗^	0.00^∗∗^
Courtesies (SF1)	96^∗^	TANC		
Assertion (SF2)	93^∗^	92^∗^	92.50^∗^	0.01^∗^
Question (SF3)	95^∗^	94^∗^	94.50^∗^	0.01^∗^
Agreement (SF4)	92^∗^	95^∗^	93.50^∗^	0.02^∗^
Denial (SF5)	TANC	79^∗^		
Direction (SF6)	TANC	TANC		
Verbal Mode-Communicative Intent (VeM-CI)	87^∗∗^	92^∗∗^	89.50^∗∗^	3.54^∗∗^
Acknowledging (CI1)	93^∗^	97^∗^	95.00^∗^	0.03^∗^
Informing (CI2)	65^∗^	TANC		
Exploring (CI3)	86^∗^	86^∗^	86.00^∗^	0.00^∗^
Deepening (CI4)	75^∗^	82^∗^	78.50^∗^	0.05^∗^
Focusing (CI5)	79^∗^	82^∗^	80.50^∗^	0.02^∗^
Temporizing (CI6)	TANC	TANC		
Attuning (CI7)	70^∗^	90^∗^	80.00^∗^	0.14^∗^
Resignifying (CI8)	100^∗^	82^∗^	91.00^∗^	0.13^∗^
Vocal Mode (VoM)	93^∗∗^	87^∗∗^	90.00^∗∗^	4.24^∗∗^
Reporting (VM1)	100^∗^	TANC		
Connected (VM2)	87^∗^	89^∗^	88.00^∗^	0.01^∗^
Declarative (VM3)	75^∗^	77^∗^	76.00^∗^	0.01^∗^
Introspective (VM4)	80^∗^	100^∗^	90.00^∗^	0.14^∗^
Emotional-Positive (VM5)	83^∗^	85^∗^	84.00^∗^	0.01^∗^
Emotional-Negative (VM6)	88^∗^	61^∗^	74.50^∗^	0.19^∗^
Pure Positive Emotion (VM7)	100^∗^	100^∗^	100.00^∗^	0.00^∗^
Pure Negative Emotion (VM8)	TANC	TANC		
Interruption Mode (IM)	81^∗∗^	92^∗∗^	86.50^∗∗^	7.78^∗∗^
Cooperative-Concurrence (IM1)	89^∗^	96^∗^	92.50^∗^	0.05^∗^
Cooperative-Assistance (IM2)	TANC	100^∗^		
Cooperative-Clarification (IM3)	100^∗^	85^∗^	92.50^∗^	0.11^∗^
Cooperative-Exclamation (IM4)	TANC	100^∗^		
Intrusive-Disagreement (IM5)	87^∗^	83^∗^	85.00^∗^	0.03^∗^
Intrusive-Floor taking (IM6)	TANC	89^∗^		
Intrusive-Competition (IM7)	TANC	100^∗^		
Intrusive-Topic change (IM8)	TANC	TANC		
Intrusive-Tangentialization (IM9)	TANC	TANC		
Neutral interruption (IM10)	93^∗^	81^∗^	87.00^∗^	0.08^∗^
Failed Interruption (IM11)	TANC	90^∗^		


*TANC, Total Agreement in the Not Coded Category; scores are expressed in percentage; ^∗^Inter-judge reliability through Cohen’s kappa (κ); ^∗∗^Inter-judge reliability through Krippendorff’s canonical agreement coefficient (Cc); κ and Cc: insufficient (lower than or equal to 60%), substantial (between 61 and 80%), satisfactory (greater than or equal to 81%).*

The CMASP reaches from high to very high intra-and inter-judge reliability for those categories expressing objective aspects of communication (the VeM-Structural Form categories) as well as for those categories based on the communicative intent (the categories of the VeM-Communicative Intent, VoM, and Interruption Mode dimension) which stimulate the subjectivity of the coder.

### CMASP Applications: Descriptive Statistics of the Subscales Trend and an Example of Coding

As it is possible to see in Table 3, by the application of the CMASP on the 20 psychotherapy sessions (for a total of 6232 speaking turns), the VeM-Structural Form dimension shows the highest percentage of codes indicating high participation in communicative exchanges through speech contents with a clear structure. Precisely, speakers mainly expressed verbalizations in the form of statements (Assertion), recognition of the truth of the other’s statements (Agreement) and requests for information (Question). A high percentage of communicative intents (VeM-CI) accompanied such structural forms, mainly characterized by asking for/providing contents (Exploring), taking the other’s viewpoint (Acknowledging), deepening contents (Deepening), Resignifying, and Attuning (even if at a lesser percentage). It expresses the typical characteristics emerging in the initial phases of psychodynamic psychotherapy, although the CMASP brings added value since it is possible to integrate information corresponding to co-occurrences of behavior in all dimensions.

**Table 3:** Descriptive statistics of the CMASP communicative modes on the definitive sample (*N* = 6232 speaking turns).

CMASP	*f*	%
Verbal Mode-Structural Form (VeM-SF)	5748	92.23
Courtesies (SF1)	52	0.90
Assertion (SF2)	3299	57.39
Question (SF3)	752	13.08
Agreement (SF4)	1516	26.37
Denial (SF5)	80	1.39
Direction (SF6)	49	0.85
Not coded	484	7.77
Verbal Mode-Communicative Intent (VeM-CI)	5171	82.97
Acknowledging (CI1)	1275	24.66
Informing (CI2)	196	3.79
Exploring (CI3)	2285	44.19
Deepening (CI4)	568	10.98
Focusing (CI5)	181	3.50
Temporizing (CI6)	26	0.50
Attuning (CI7)	227	4.39
Resignifying (CI8)	413	7.99
Not coded	1061	17.03
Vocal Mode (VoM)	3832	61.49
Reporting (VM1)	10	0,26
Connected (VM2)	1521	39.69
Declarative (VM3)	214	5.58
Introspective (VM4)	151	3.94
Emotional-Positive (VM5)	965	25.18
Emotional-Negative (VM6)	588	15.34
Pure Positive Emotion (VM7)	333	8.69
Pure Negative Emotion (VM8)	50	1.30
Not coded	2400	38.51
Interruption Mode (IM)	1144	18.36
Cooperative-Concurrence (IM1)	314	27.45
Cooperative-Assistance (IM2)	32	2.80
Cooperative-Clarification (IM3)	83	7.26
Cooperative-Exclamation (IM4)	18	1.57
Intrusive-Disagreement (IM5)	50	4.37
Intrusive-Floor taking (IM6)	185	16.17
Intrusive-Competition (IM7)	94	8.22
Intrusive-Topic change (IM8)	19	1.66
Intrusive-Tangentialization (IM9)	3	0.26
Neutral interruption (IM10)	286	25.00
Failed Interruption (IM11)	60	5.24
Not coded	5088	81.64


During sessions, a fairly high percentage of VoM, spreading the underlying intentions apart from the verbal content, enriched speakers’ speech. Compared to the expressed content, the voice of participants above all presented an elaborative speech in connection to oneself and oriented to the other (Connected); moreover, it transmitted positive/negative emotional states (Emotional-Positive and Emotional-Negative), positive non-verbal emotions (Pure Positive Emotions) and expressed certainty and conviction (Declarative), filling contents of new meanings.

Finally, the IM dimension shows the lowest percentage of codes compared to the 6232 speaking turns considered. As we mentioned, these modes represent an interactive aspect of communication as violations of the other participant’s communicative space by an interrupter. Therefore, such a percentage do not indicate a negative aspect but, on the contrary, it expresses good self-regulation and coordination capacities of both participants during communicative exchanges. Generally, participants interrupted to show concurrence (Cooperative-Concurrence), neutrally take the floor (Neutral Interruption), or intrusively develop the topic of the current speaker (Intrusive-Floor taking). Moreover, they interrupted generating a battle to take the floor and express one’s speech (Intrusive-Competition), or they could interrupt to understand the other’s speech (Concurrence-Clarification).

The separate analysis of the CMASP categories aims to show the trend of each categorical system within the instrument. The integration of the communicative modes of the different dimensions occur at the interpretative level according to the values that these assume in line or not with the expected distributions; this makes it possible to determine different communication profiles that participants carry out. Assume that a speaker 1 shows the following communicative modes that are higher to the expected distribution: Assertion (VeM-SF), Exploring (VeM-CI), and Emotional-Positive (VoM). Moreover, assume that a speaker 2 shows the following communicative modes that are higher to the expected distribution: Assertion (VeM-SF), Exploring (VeM-CI), Emotional-Negative (VoM), and Intrusive-Floor taking (IM). It is possible to notice that, although both speakers use the same verbal communication modes, non-verbal modes convey speech in different ways, determining two distinct communication profiles. Speaker 1, indeed, refers to a certain state of things (Assertion) by reporting his/her inner experience (Exploring) that is modulated by a positive emotional state (Emotional-Positive). Speaker 2, on the other hand, interrupts intrusively to take the floor (Intrusive-Floor taking IM) reporting his/her inner experience filled with negative emotions (Emotional-Negative).

Considering that each patient assumes an interactive role with his/her therapist and that for each one it is possible to detect the specific communicative modes, it results that we can have a detailed and “individualized” profile for the patient, therapist, and their unique interaction.

It is important to underline that some speaking turns were not coded due to the sensitivity of the classification system in coding certain communicative behaviors (e.g., VoMs cannot be detected in a speech less than 2 s). Moreover, some categories showed a lower percentage than others, not because they were not present, but because the CMASP attributes a predominant communicative mode to a speaking turn for most of the dimensions (VeM-SF, VeM-CI, VoM). As we mentioned, this classification system micro-analyzes each speaking turn which can be segmented when changes occur in the communicative modes. Therefore, although these categories (e.g., Courtesies, Denial, Direction, Temporizing, Reporting) could occur in a segment at a micro level, the attribution of the predominant category decreased their probability of being coded at a speaking turn level. On the contrary, other categories (e.g., Pure Negative Emotion, Cooperative-Assistance, Cooperative-Exclamation, Intrusive-Topic change, Intrusive-Tangentialization) could present a lower percentage, although not being based on the predominance coding procedure, due to the specific characteristics of the communicative interactions with depressed patients.

Hereunder, we present an example of the CMASP coding to show its capability to analyze the complexity of the psychotherapeutic exchange and giving information about the psychotherapy process (Table 4). Such a segment is extrapolated from the second session of psychodynamic psychotherapy, belonging to the final sample of seven cases, and it is related to communicative exchanges between a male patient with depressive symptomatology and the female therapist.

**Table 4:** Illustration of the CMASP coding.

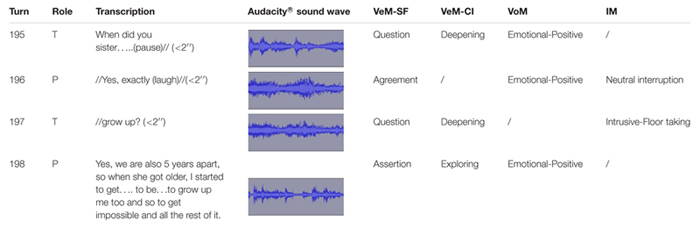

*T, Therapist; P, Patient; VeM-SF, Verbal Mode-Structural Form; VeM-CI, Verbal Mode-Communicative Intent; VoM, Vocal Mode; IM, Interruption Mode. /, indicates the not-coded communicative behaviors. //, indicates the speaking turn interruption. (<2′′), indicates speech less than 2 s.*

A trained coder, using both the audio recording and the verbatim transcript, realized the classification of patient and therapist’s verbal and extra-linguistic communicative modes. Following the coding manual, he used the verbatim transcript to detect the different structural forms and communicative intents of verbal modes turn-by-turn. Moreover, he employed the transcript as support to note the extra-linguistic modes, emerging in each therapist and patient’s speaking turn, detected by a careful listening of the audio recording. If two or more communicative modes of the same CMASP dimension occurred in a speaking turn, the coder assigned the predominant one according to the coding rules of the manual.

Table 4 represents an illustration that shows the added value of the CMASP by integrating the information from several components. As it is possible to notice, in speaking turn no. 195 and no.197, the therapist asks for information (VeM-SF: Question) with the intent of deepening (VeM-CI: Deepening) “stimulated” by the patient’s previous speech. The therapist expresses this through a positive emotion (VoM: Emotional-Positive) in speaking turn no. 195 since her speech affects the listener as filled with curiosity. According to the coding procedures, the CMASP cannot code VoMs in speaking turns less than 2 s unless they express emotional states. In speaking turn no. 195, therapist pauses her speech for a moment arousing uncertainty in the patient about her intention to continue to speak. Consequently, in speaking turn no. 196, the patient starts to speak without a real interruption (IM: Neutral interruption) to recognize the truth of the therapist’s statement (VeM-SF: Agreement). He modulates his speech through a laugh (VoM: Emotional-Positive), synchronizing with the positive emotional state expressed by the therapist. In speaking turn no. 197, faced with such communication of agreement supported by positive emotion, the therapist intrusively interrupts the patient to regain the floor (IM: Intrusive-Floor taking) with the intent to continue her question about the previous speech (VeM-SF: Question; VeM-CI: Deepening). In speaking turn no. 198, the patient starts to speak in a coordinated way, referring to a certain state of things (VeM-SF: Assertion), to provide the information required by the therapist and giving new contents in the form of past experiences (VeM-CI: Exploring).

Such a speaking turn would be segmented due to the initial structural form of agreement (“Yes”). However, Assertion represents the predominant VeM-SF expressed by the patient for the rest of the speech and, for this reason, it can be attributed as the only code to the entire speaking turn. Finally, when the patient talks about his adolescence and the relationship with the sister, his speech affects the listener of the therapeutic exchange as filled with tenderness (Emotional-Positive).

The segment shows positive communicative exchanges between the therapist and the patient in which the two participants are emotionally synchronized. The previous patient’s speech stimulates the emerging of a positive emotional state in the therapist which, at the same time, transmits to the patient the recognition of his experience and sustains the therapist herself in deepening the content referred. In turn, the patient emotionally and cognitively recognizes what the therapist expresses in the therapeutic relationship and transmits receptiveness to this last one. All this generates a climate of sharing and closeness which enables the therapist to reach the internal reality of the patient who, in turn, feels understood and supported in exploring his experience. In this case, the emotional climate helps the patient to get in touch with his emotions and legitimates him to attribute new meaning to his internal world through the sharing with the therapist. Instead, the disruptive interruption of the therapist sustains the patient in maintaining the emotional and relational balance, representing a typical problem of patients with depressive symptoms.

This illustration represents an example that shows the capacity of the CMASP to provide multiple and concurrent information about the intersubjective processes implemented by the therapist and patient during communicative exchanges. What emerges is a multi-level complexity in which the mutual regulation process occurs according to multiple and simultaneous directions (verbal–verbal, verbal–non-verbal, non-verbal–verbal, non-verbal–non-verbal). All this allows us to comprehend that these aspects of communication (content, voice, and interruptions) interweave during the co-construction of the therapeutic interaction and they cannot be considered as independent elements. Naturally, the complexity and dynamicity of the psychotherapeutic exchange make difficult the complete knowledge of what occurs within the psychotherapy setting, but the CMASP provides a deeper understanding of the internal reality of each participant and their mutual regulation during the psychotherapy session. Therefore, as an integrated system, the CMASP enables the professionals and researchers to obtain consistent information about some fundamental components of communication and the way they affect the co-construction of meanings and orient the psychotherapy process.

## Conclusion

The purposes of this study were, on the one hand, to introduce the building of the Communicative Modes Analysis System in Psychotherapy (CMASP) and its constituent dimensions and categories underlining its ability in detecting and coding multiple aspects of communication in psychotherapy simultaneously and, on the other hand, presenting its early reliability psychometrics for both inter-and intra-rater values. Inspired by the process of convergence of natural and human science, we developed the CMASP to overcome the limits of the psychotherapy research -which investigates and theorizes the components of communication as in polar opposition- and trying to interpret some fundamental elements of therapeutic exchanges (verbal, vocal, and interruption behaviors) as an integrated and interactive system through a comprehensive theory, derived from the linguistic field.

As the CMASP is developed within the mixed methods framework by building a qualitative system that is quantitized (Sandelowski et al., 2009), it shows an increased incremental validity which ensures the qualitative/quantitative dimensions of functioning. The structure of the CMASP as a coding system applicable to both therapist and patient, as well as the possibility of detecting a predominant encoding at a speaking turn level, allow overcoming the limits of many instruments and realizing comparative and sequential analysis of communicative modes implemented by both participants during the psychotherapy process, increasing the knowledge about their evolution. In particular, the instrument permits to classify verbal and non-verbal aspects connected to the effectiveness of psychotherapy and identifying the communication profiles that contribute to the process of change in patients.

Given its high reliability at the global, dimensional, and categorical level, the CMASP represents an effective instrument providing researchers and professionals with a single classification system, able to give multiple and concurrent information about patient-therapist communicative exchanges and their evolution during a psychotherapy session. Moreover, given its flexibility, this classification system allows focusing the knowledge on a specific area of communication. Precisely, the instrument can be used as a single system permitting to monitor simultaneously verbal and non-verbal changes bound up with psychotherapy, especially when it is applied together with other instruments (e.g., self-reports, clinical reports) to improve the incremental validity of the effectiveness measure. Alternatively, as the verbal and non-verbal dimensions of the CMASP can also be applied separately, the instrument can provide an objective measure of change -starting from the qualitative modes of relational exchange- in case of disorders (depression, ADHD, BPD) with marked non-verbal behaviors.

On the one hand, it could represent a useful instrument for researchers to increase the knowledge about what is occurring within the psychotherapy process reducing its complexity and, on the other hand, it could support the clinician in comprehending the patient functioning and improving the interventions tailored to each specific therapeutic interaction. Concerning to researchers, for example, the CMASP could allow them to deepen the knowledge about the interaction of communicative modes with other constructs (e.g., therapeutic alliance, attachment patterns), or different disorders (e.g., anxiety, eating disorders), or changes in patient’s symptoms after and before the treatment. Concerning the clinicians, our final purpose would be to provide them with an instrument they will be able to internalize with practice, without the need for the physical support of audio recordings and verbatim transcripts, integrating it with their skills for sustaining the interaction with the patient and the psychotherapy process. For example, by recognizing the non-verbal communication underlining the expressed content (e.g., an elaborative speech, a positive emotional state, an interruption to clarify or to disrupt), the clinician may draw information about the coherence between the verbalized content and non-verbal modes associated, about the patient’s resistance, or the internalized meaning he/she expresses behind and with words. In this way, the clinician can calibrate with more efficacy his/her intervention toward the patient.

Based on decades of studies on communication in the field of psychotherapeutic research, the CMASP attempts to contribute to understanding the complexity of this field by deepening the dynamic process of co-construction of meanings during patient–therapist communicative exchanges. The development of such a classification system showed the difficulty in coping with methodology problems in the communication study. These preliminary results come from the application of coding and counting approaches belonging to the tradition of research on communication, but we aim to integrate these as a part of a system in interaction in future studies (Peräkylä, 2004).

Firstly, since this paper is an early introduction of the classification system building and its psychometric properties, we aim to focus on its validation in future research. Moreover, convergent and discriminant validity studies are not available, but the CMASP segmentation procedure -elaborated through the CIS-R one- will allow performing correlational studies of validity between the communicative modes and the therapeutic alliance as well as internal correlation analyses among the categories, in future research. Finally, even though some categories of the CMASP show a low percentage, this is not a negative aspect as it may be due to the specificity of the sample (patients with depressive symptomatology), on the contrary, it provides information about the communicative characteristics of certain types of psychotherapeutic interactions, increasing the knowledge on this type of patients. Given the instrument flexibility, we aim to extend its application to other psychotherapy sessions, patients and, mostly, disorders. It is possible, for example, that a category like VOM-Declarative, with a low percentage in depressed patients, could characterize other types of disorders (e.g., narcissistic patients) predominantly.

Although the CMASP seems to solve the problem of understanding the communicative exchanges in psychotherapy through the pragmatic function of language as a global theory -increasing knowledge about what occurs during the interaction between the patient and therapist- the insubstantiality of certain distinctions between verbal and non-verbal aspects makes further studies necessary from an interdisciplinary standpoint. The CMASP development was based on the observation of psychotherapies conducted by just one therapist. At first, such a choice was made to reduce variability in the pilot research, but we know this decision could affect data because of the personal style of the therapist, or biases, or the individual communicative trends. For these reasons, in future research, it would be useful to consider the observation of more therapists to extend, improve and confirm the communicative modes analyzed. Furthermore, we observed only psychotherapies conducted by a female therapist. In future research, it would also be useful to observe psychotherapies conducted by a male therapist to verify if gender may affect the use of specific communicative modes (e.g., to examine if a female therapist may use more emotional communicative modes than a male therapist). We selected patients according to depressive symptomatology, but the purpose for future research is to extend the CMASP application to other types of disorders (e.g., anxiety, emotional dysregulation, obsessive–compulsive behaviors, eating disorders and so on) for creating a diagnostic classification system with established norms, or trends, for each diagnostic category. Finally, it would be useful to integrate the observation of video recording to extend the richness of communication in psychotherapy with other non-verbal components (e.g., facial expression or body movement observation).

## Ethics Statement

We developed the CMASP at the Dynamic Psychotherapy Service belonging to the Interdepartmental Laboratories for Research and Applied Psychology (LIRIPAC), a recognized research center of the University of Padua (Italy). The ethics committee of psychology faculty of the University of Padua approved the collection of the research material (informed consents, the audio recording of sessions, and confidentiality modes of procedures) which followed the ethical guidelines and procedures of the LIRIPAC, based on the Italian law about privacy and confidentiality (n. 196/03). We discussed the specific research practice and ethical procedure of this investigation with the Director of the Centre who approved them before the research began in 2016. We followed the ethical standards for research outlined in the Ethical Principles of Psychologists and Code of Conduct (American Psychological Association [APA], 2017). Therefore, we assured confidentiality by replacing the participants’ personal information. As for listening to the audio recordings, we guaranteed confidentiality not providing personal data of the speakers to the trained coders who were in charge of their listening and transcription. We did not award incentives, and we emphasized voluntary participation. In line with the Declaration of Helsinki, we collected the informed consents of the therapist (verbal consent) and each patient (written consent), finalized to research aims, before realizing data collection and audio recording. In other words, we conducted the study after the end of the psychotherapy treatments.

## Author Contributions

LDG documented, designed, drafted, and wrote the manuscript. Moreover, he trained and supervised the coders as well as he carried out statistical analyses. SS supervised the sample recruitment and the statistical analyses. MA supervised the method and procedure sessions as well as statistical analyses. SS and MA revised the manuscript for theoretical and intellectual content. Finally, all authors provided final approval of the version to be published.

## Conflict of Interest Statement

The authors declare that the research was conducted in the absence of any commercial or financial relationships that could be construed as a potential conflict of interest. The reviewer PDC declared a shared affiliation, with no collaboration, with several of the authors, LDG, SS, to the handling Editor at the time of review.
