# Supplementary material for: The Communicative Modes Analysis System in Psychotherapy From Mixed Methods Framework: Introducing a New Observation System for Classifying Verbal and Non-verbal Communication
Source: Front Psychol. 2019 Apr 10;10:782. doi: 10.3389/fpsyg.2019.00782 (PMC6467955; doi:10.3389/fpsyg.2019.00782)
Supplement: Supplementary file 1 [file Table_1.pdf]

## *Supplementary Material*

# The Communicative Modes Analysis System in Psychotherapy from Mixed Methods framework: Introducing a New Observation System for Classifying Verbal and Nonverbal Communication

**Luca Del Giacco[1,2*], Silvia Salcuni[2], and M. Teresa Anguera[3]**
[1]Department of Social Psychology and Quantitative Psychology, University of Barcelona, Barcelona, Spain.
[2]Department of Developmental Psychology and Socialization, University of Padua, Padua, Italy.
[3]Faculty of Psychology, Institute of Neurosciences, University of Barcelona, Barcelona, Spain.

***Correspondence**: Dr. Luca Del Giacco: lucadelgiacco@gmail.com

## Appendix I. Description of the CMASP dimensions and categories

| Dimension | Categories | Description | Code |
|---|---|---|---|
| Verbal Mode-Structural Form (VeM-SF)<br><br>It concerns the formal structure of the speech by which the speaker expresses the verbal mode (it corresponds to the propositional component of the speaker's speech). | Courtesies | Speaker's speech is in the form of terms expressing receptiveness to the communication according to social conventions (e.g., *"Good morning"*, *"Goodbye"*, *"Thank you"*, *"Your welcome"*). | SF1 |
| | Assertion | Speaker's speech expresses something he/she considers true, or it refers to a specific state of the things (e.g., *"I feel empty"*, *"I can hardly concentrate"*). | SF2 |
| | Question[1] | Speaker's speech is in the form of a request for specific information (e.g., "*Would you like to tell me the problem?"*, *"And this laziness hum for example in what…"*, *"So, you're not Italian…"*). | SF3 |
| | Agreement | Speaker's speech recognizes the truth of the other's statement (e.g., *"Mm-hm"*, *"Right"*, *"Yes"*, *"Of course"*, *"Perhaps"*, *"All right"*). | SF4 |
| | Denial | Speaker's speech refuses or rejects to recognize the truth of something said by the other (e.g., *"No"*, *"In no way"*, *"Absolutely no"*). | SF5 |

| | | | |
|---|---|---|---|
| | Direction | Speaker's speech encourages the listener towards cognitive, emotional, or behavioral actions by guiding the other's behavior (e.g., *"Tell me what's wrong"*). | SF6 |
| Verbal Mode-Communicative Intent (VeM-CI)<br><br>It concerns the underlying intention of the speaker's speech (it corresponds to the performative component of communication). | Acknowledging | Speaker's communicative intent is to take the other's point of view about the experience of this last one, not presuming a specific knowledge of the other's experience but the speaker's one only (e.g., *"Mm-hm"*, *"Ok"*; *"Exactly"*, *"Mh"*, *"Right"*, *"Good morning"*, *"Goodbye"*, *"Thank you"*, *"Your welcome"*). | CI1 |
| | Informing | Speaker's communicative intent is to supply (or request for), information about the here and now of psychotherapy in the form of data, facts, resources, theory, and assessment parameters. The information may be specifically related to the counseling process, therapist behavior, or arrangements (time, place, fee, and so on), (e.g., T: *"We'll meet once again, and then we'll take stock of the situation"*). | CI2 |
| | Exploring | Speaker's communicative intent is to ask for information about knowledge, events, feelings, or about the causes of content or behavior (e.g., T: *"Would you like to tell the reason you are here?*"; T: *"How was your move to Padua?"*). Moreover, the speaker can provide the information required by the other focusing on knowledge, events or feelings (e.g., P: *"My parents are divorced"*), or he/she can give new contents in the form of stories as well as descriptions of past or present experiences (e.g., P: *"When I was a child, I liked to sleep with my parents"*). Finally, he/she can describe a feeling or emotional state (e.g., P: *"I've no energy and I always feel sad"*). | CI3 |
| | Deepening | Speaker's communicative intent is to deepen the description, presentation, or discovery of some contents. He/she can realize it: a) verifying the truthfulness of an assertion made by the other which is questioned (e.g., P: *"I got so mad when he said to me those words, but you I'm fine on my own"* – T: *"So, don't you care of what the others say?"*); b) correcting the comprehension of the other (e.g., T: *"If I've understood correctly, it sounds like your problem is due to relationships"* – P: *"No, the problem is only with my mother"*); c) corroborating something stated (an opinion, facts, or new contents which are given or requested by the other) (e.g., T: *"So, you're one of the most aged"* – P: *"Yes, I was selected for my age"*); d) requesting for information about the content of the other's communication (e.g., P: *"I've* | CI4 |

| | | |
|---|---|---|
| | *called him many times but…nothing"* – T: *"In other words, hasn't he called you back anymore?"*). | |
| Focusing | Speaker's communicative intent is to direct the attention and efforts towards a specific topic of conversation. The speaker can realize it: a) introducing/addressing a topic (e.g., P: *"Well, I would like to start with the reasons"*); b) returning to a topic (e.g., T: *"So, getting back to what we were talking about"*); c) summarizing a content (e.g., T: *"Today we have spoken about many things"*); d) defining the limits of a given content (e.g., T: *"I'd like to focus on the relationship with your boyfriend"*). | CI5 |
| Temporizing | Speaker's communicative intent is to assume a suspended position as regards the other's communication. This allows the speaker to get in touch with his/her thoughts and feelings or, on the contrary, to avoid facing the requests of the previous speech, momentarily (e.g., T: *"How did you feel?...How.."* – P: *"How I felt…"*). | CI6 |
| Attuning | Speaker's communicative intent is to understand or be understood by the other. He/she realizes this: a) verifying his/her comprehension with a careful examination of what he/she understood about the other's communication (e.g., T: *"Let me get this straight, you're telling me your mom doesn't know you smoke"*); b) telling the other how his/her actions or thoughts are being understood (e.g., T: *"In other words you think your mood is due to your parents' divorce"*); c) communicating to the other that his/her actions or thoughts are understood (e.g., T: *"Now I see, in other words, you're a sophomore in University"*). Moreover, to express attuning, the speaker can harmonize with the other showing an emotional connection to his/her reality (e.g., T: *"I imagine it's a difficult situation"*). Finally, the speaker can perform such a communicative intent by providing feedback: a) validating or discouraging the other's behaviors, meanings or feelings (e.g., T: *"Don't worry, go on"*); b) showing the other's affections or telling the emotional impact that the other had on the speaker (e.g., T: *"I'm making you angry"*). | CI7 |
| Resignifying | Speaker's communicative intent is: a) to offer a new perspective on content (e.g., T: *"Maybe, there is also the fear of not being understood"*); b) to connect contents, to one another (e.g., P: *"I realize that I tend to get angry at my boyfriend like my father"*); c) to recognize or establish a psychological working model (e.g., T: *"You have a very rigid way of facing things* ); d) to | CI8 |

| | | | |
|---|---|---|---|
| | | question a content (e.g., T: *"Well, but it seems you're afraid to understand you can do it on your own"*). | |
| Vocal Mode (VoM)<br><br>It concerns the underlying intentions of the speaker's speech associated with both a peculiar acoustic parameters combination (tone, intensity, duration, and timbre) and a specific way the speaker's speech affects the listener of communicative exchange, apart from the verbal content. The "emotional" categories building follows the principle of universal emotions recognition. | Reporting | The listener has the impression of a detached speech emitted by the speaker like he/she is reporting, narrating, or exploring contents without any emotional involvement. Speaker's voice seems to attribute a detached quality to speech; moreover, an emotional disconnection and (or) emotional distance seems to characterize what is being said. A typical vocal parameter of this category is the repetitive prosody (concerning the tone) which, in turn, presents agogic accent (concerning the tone) and high variation in the dynamic (concerning the intensity). Finally, the speech is usually characterized by fluid pace (concerning the duration). | VM1 |
| | Connected | The listener has the impression of an elaborative speech emitted by the speaker and oriented to the other. Speaker's voice seems to attribute to speech the quality of being connected and (or) attuned to him/herself as well as to the other, giving this last one space for intervening. The distinctive vocal parameters of this category are the anti-cadence, characterizing the end of the sentence expressed, and agogic accent (concerning the tone) with a soft-vocal attack (concerning the duration). Finally, the pace may present pauses and loss of fluidity (e.g., extensions, repetitions and so on), concerning the duration. | VM2 |
| | Declarative | The listener has the impression of a secure, instructive, engaged or convinced talk emitted by the speaker. Speaker's voice seems to attribute the quality of certainty and conviction to the speech like he/she is instructing (or explaining to) the other, or like he/she seems very sure of (engaged in) what he/she is saying. The other has a little space for intervening. The peculiar vocal parameters of this category are the suspended or anti-cadence, characterizing the end of the sentence expressed, as well as agogic and (or) dynamic accent (concerning the tone) with a hard-vocal attack (concerning the intensity). The pace is usually fluid (concerning the duration) while the intensity may present an average volume increased (concerning the intensity). | VM3 |
| | Introspective | The listener has the impression of an introverted speech emitted by the speaker. Speaker's voice seems to attribute to speech the quality of being directed toward her/himself like he is connected with her/his internal world | VM4 |

| | | |
|---|---|---|
| | or in a dialog with her/himself. The distinctive vocal parameters of this category are an average volume decreased and dynamics decrescendo (concerning the intensity). Sometimes, this vocal mode may present a reduced speed and long pauses (concerning the duration). | |
| Emotional-Positive | The listener has the impression of a positive-emotional speech emitted by the speaker. Speaker's voice seems to attribute positive affection and (or) positive emotional strength to speech. Such a vocal mode expresses the speaker's positive emotion (e.g., cheerfulness, happiness, sweetness, excitement, charm, understanding) modulating the verbal component of the speech (e.g., a laugh, shrill or sweet voice can accompany it) or, on the contrary, the effort to contain the emotion. A typical parameter of this category is the timbre, characterizing speech with variation in color and bright (Clear/Bright and Clear/Opaque), associated with changes in the sounding system (e.g., the shape that mouth assumes when someone smiles) expressing positive affection. Moreover, this category is often associated with a soft-vocal attack. | VM5 |
| Emotional-Negative | The listener has the impression of a negative-emotional speech. Speaker's voice seems to attribute the quality of negative emotion and (or) negative emotional strength to speech. Such a vocal mode expresses the speaker's negative emotion (e.g., anger, sadness, fear, tension) modulating the verbal aspect of the speech (e.g., sobbing, broken voice, trembling voice, snort can accompany it) or, on the contrary, the effort to contain the emotion. A typical parameter of this category is the timbre, characterizing speech with variation in color and bright (Clear/Bright, Dark/Bright, and Dark/Opaque), associated with changes in the sounding system (e.g., the nasal congestion when someone cries, or the tension of the vocal cord when someone is nervous) expressing negative affection. Moreover, this category is often associated with an increased volume and (or) a not fluid pace. Finally, an increased volume and/or a no fluid pace is often associated with this category. | VM6 |
| Pure Positive Emotion | Speaker's voice quality expresses a positive emotional state (e.g., doing a half-smile, laughing) without uttering any verbal content. The speaking turn is characterized only by vocalizations, due to changes in the sounding system, expressing a positive emotion and no utterance precedes or follows. | VM7 |

| | | | |
|---|---|---|---|
| | Pure Negative Emotion | Speaker's voice quality expresses a negative emotional state (e.g., crying, sighing) without uttering any verbal content. The speaking turn is characterized only by vocalizations, due to changes in the sounding system, expressing a negative emotion and no utterance precedes or follows. | VM8 |
| Interruption Mode (IM)<br><br>It concerns the interrupter's behaviors, implemented to take the floor (successfully or not), for supporting or hindering the communicative flow of the current speaker. These modes analyze the potential violations of the transition relevance place (TRP) by the interrupter, as well as the impact the interruption has on the other participant and the "reaction" that this last one implements towards the interrupter. Each mode is defined by the moment and way the interrupter takes the floor as well as by the purpose of his/her interruption. | Cooperative-Concurrence | This kind of interruption enables the interrupter to show agreement, validation, understanding, compliance, or support to the current speaker. Sometimes, the interruption also aims to extend or elaborate on the idea presented by the speaker. | IM1 |
| | Cooperative-Assistance | This interruption mode enables the interrupter to sustain the current speaker by providing a word, phrase, sentence, or idea when the interrupter perceives the current speaker needs help. | IM2 |
| | Cooperative-Clarification | The interrupter usually implements this kind of interruption mode with the intention to understand the message sent by the current speaker. The ultimate goal of the interruption is to make sure that the current speaker clarifies or explains a previously expressed piece of information the listener is dubious. In other words, when the listener is unclear about a piece of information the current speaker has just expressed, he/she interrupts this last one to request clarifications. | IM3 |
| | Cooperative-Exclamation | This mode is implemented by the interrupter to show rapport as well as coparticipant involvement by expressing surprise to the previous utterance of the speaker. | IM4 |
| | Intrusive-Disagreement | This mode occurs when the interrupter intervenes to show disagreement about what the speaker is saying and wants to correct or express his/her opinion immediately. | IM5 |
| | Intrusive-Floor taking | This kind of interruption mode occurs when interrupter intervenes to develop the topic of the current speaker by taking over the floor from this last one. Generally, the interrupter does not intend to change the topic of the speaker, but only express his/her opinion, idea, thoughts, by taking the floor. | IM6 |
| | Intrusive-Competition | This kind of interruption is characterized by a simultaneous speaking in which both participants interrupt each other to complete their speech, generating a real fight for the floor. In such an interruption, the one who first interrupted manages to take the floor and to prevent the other to end his/her speech. | IM7 |

| | | |
|---|---|---|
| Intrusive-Topic change | It occurs when the interrupter intervenes to change the topic cutting the speech of the current speaker. The interrupter is somewhat more aggressive than in the floor-taking situation because he/she must accomplish the task of changing the topic. | IM8 |
| Intrusive-Tangentialization | In this kind of interruption, the listener interrupts to summarize the information sent by the current speaker, reflecting his/her awareness. In other words, since the interrupter does not want to listen to the same information repeatedly, then he/she intervenes to summarize one or more pieces of the previously expressed information; in this way, he/she minimizes the message sent by the current speaker. Tangentialization prevents the interrupter from listening to an unwanted piece of information because either the information has been presented previously or the listener through other channels already knows the information. | IM9 |
| Neutral interruption | This kind of interruption mode is neither cooperative nor intrusive (it does not violate the principles of turn change). It occurs when the speaker pauses or stops the talk, creating uncertainty about his/her intention to continue the speech and, consequently, the interrupter takes the floor and starts to talk. The central aspect of this kind of IM is that the speaker's speech appears incomplete due to his/her stop. | IM10 |
| Failed Interruption | A simultaneous speech characterizes the present interruption mode, but there is no turns exchange as in the IM Intrusive-Competition. It occurs when the listener tries to intervene interrupting, but he/she stops before finishing the intruding speech since the current speaker continues talking. This last one ignores the interrupter and continues talking until he/she finishes. In other cases, the interrupter stops before completing his/her intruding speech since he/she understands the speaker wants to continue talking. | IM11 |

*Note.* [1]Question represents a complex category resulting from the combination of different aspects at the same time (social action, sequential position, and turn-design features; Stivers and Rossano, 2013). Different forms of questions were considered, indicating different degrees of disparities in the reciprocal participants' knowledge and conveying the relationship between them (Park, 2012).

## References

Park, I. (2012). Asking different types of polar questions: The interplay between turn, sequence, and context in writing conferences. *Disc. Stud.* 14:5, 613–633. doi:10.1177/1461445612454077

Stivers, T., and Rossano, F. (2013). "Mobilising response in interaction: a compositional view of questions," in *Questions: Formal, functional and interactional perspectives*, ed. J.-P. de Ruiter (Cambridge: Cambridge University Press), 58–80.
